# Lithium ameliorates tubule-interstitial injury through activation of the mTORC2/protein kinase B pathway

**DOI:** 10.1371/journal.pone.0215871

**Published:** 2019-04-19

**Authors:** Douglas E. Teixeira, Diogo B. Peruchetti, Leandro S. Silva, Rodrigo P. Silva-Aguiar, Morgana B. Oquendo, João Luiz Silva-Filho, Christina M. Takiya, José Henrique Leal-Cardoso, Ana Acacia S. Pinheiro, Celso Caruso-Neves

**Affiliations:** 1 Instituto de Biofísica Carlos Chagas Filho, Universidade Federal do Rio de Janeiro, Rio de Janeiro, RJ, Brazil; 2 Instituto Superior de Ciências Biomédicas, Universidade Estadual do Ceará, Fortaleza, CE, Brazil; 3 Instituto Nacional de Ciência e Tecnologia em Medicina Regenerativa, INCT-Regenera, Conselho Nacional de Desenvolvimento Científico e Tecnológico/MCT, Rio de Janeiro, Brazil; Universidade de Sao Paulo, BRAZIL

## Abstract

Tubule-interstitial injury (TII) is a critical step in the progression of renal disease. It has been proposed that changes in proximal tubule (PT) albumin endocytosis plays an important role in the development of TII. Some reports have shown protective effects of lithium on kidney injury animal models that was correlated to proteinuria. We tested the hypothesis that lithium treatment ameliorates the development of TII due to changes in albumin endocytosis. Two experimental models were used: (1) TII induced by albumin overload in an animal model; (2) LLC-PK1 cells, a PT cell line. Lithium treatment ameliorates TII induced by albumin overload measured by (1) proteinuria; (2) collagen deposition; (3) area of tubule-interstitial space, and (4) macrophage infiltration. Lithium treatment increased mTORC2 activity leading to the phosphorylation of protein kinase B (PKB) at Ser473 and its activation. This mechanism enhanced albumin endocytosis in PT cells, which decreased the proteinuria observed in TII induced by albumin overload. This effect did not involve changes in the expression of megalin, a PT albumin receptor. In addition, activation of this pathway decreased apoptosis in LLC-PK1 cells, a PT cell line, induced by higher albumin concentration, similar to that found in pathophysiologic conditions. Our results indicate that the protective role of lithium treatment on TII induced by albumin overload involves an increase in PT albumin endocytosis due to activation of the mTORC2/PKB pathway. These results open new possibilities in understanding the effects of lithium on the progression of renal disease.

## Introduction

Lithium salts have been used largely to treat mood disorders, including mania and depression [1,2]. However, this therapy is compromised due to induction of nephrotoxicity after long-term treatment in both animal models and human patients [1–5]. On the other hand, some reports showed that lithium treatment attenuated acute kidney injury (AKI) induced by gentamicin, cisplatin, lipopolysaccharide (LPS), and ischemia/reperfusion (IR) in animal models [6–8]. This protective effect of lithium was associated with action on the cortical tubular segments, indicating that lithium modulates proximal tubule (PT) function and tubule-interstitial injury (TII).

One possible cause of TII can be correlated to albumin overload in PT followed by an increase in glomerular permeability to plasma albumin [9–13]. Albumin overload promotes modifications in the cellular machinery mediating PT albumin reabsorption, which has been described to induce TII and to contribute to genesis of proteinuria [9–13]. In this context, some studies have proposed that short-term lithium treatment reduces proteinuria in different animal models of renal disease [14–16]. However, the role of lithium on PT albumin reabsorption and its correlation with the development of TII have still to be determined.

Albumin reabsorption in PT cells occurs by receptor-mediated endocytosis; megalin is the main receptor involved in this process [12,13,17]. Previous studies showed that megalin expression is decreased when PT cells are exposed to the higher albumin concentration found in pathophysiologic conditions [18–20]. It was proposed that megalin expression is a sensor for the development of TII induced by overload of albumin in PT, connecting changes in albumin concentration in the lumen of PT cells with intracellular pathways [18,19].

Previous studies have shown that there is a strict correlation between protein kinase B (PKB) activity and albumin endocytosis in PT cells, which is involved in TII induced by albumin overload [18,19,21–23]. Previously, our group showed that a higher albumin concentration decreases megalin expression and albumin endocytosis, leading to inhibition of PKB activity and, consequently, induction of cell death [18]. It is well known that PKB activation depends on the phosphorylation of 2 residues, serine 473 (Ser473) and threonine 308 (Thr308), by mammalian target of rapamycin complex 2 (mTORC2) and phospholipid dependent kinase 1 (PDK1), respectively [24]. It was shown that a higher albumin concentration induces PKB inhibition through a decrease in mTORC2 activity [19]. Interestingly, it has been shown that lithium increased PKB activity in different cell types [25–29]. Furthermore, lithium treatment reduces apoptosis in cortical tubular segments in an AKI model induced by injection of LPS [6].

Based on these observations, we can postulate that lithium treatment could modulate TII development due to changes in the machinery mediating albumin endocytosis. To test this hypothesis, we verified the effect of lithium on an animal model of TII induced by albumin overload. We observed that lithium treatment partially prevents the development of TII induced by albumin overload. This effect involves modulation of the mTORC2/PKB pathway and albumin endocytosis. These results open new possibilities in understanding the effects of lithium on renal disease.

## Materials and methods

### Materials and reagents

Bovine serum albumin (BSA), BSA conjugated to fluorescein isothiocyanate (BSA-FITC), lithium carbonate, sodium chloride, potassium chloride, magnesium chloride, calcium chloride, sodium orthovanadate, sodium pyrophosphate, sodium fluoride, sodium β-glycerophosphate, sodium azide, sodium carbonate, sodium hydroxide, ammonium persulfate, glycine, D(+)-glucose, Triton X-100, Tween 20, 4-morpholinopropanesulfonic acid (MOPS), EGTA, HEPES, periodic acid-Schiff reagent, Sirius red, Harry’s hematoxylin, streptavidin-peroxidase, Folin and Ciocalteu’s phenol reagent, protease inhibitor cocktail (no. I3786), tetramethylethylenediamine, acrylamide, bromophenol blue, 2-mercaptoethanol, Ponceau S, and phenylmethylsulfonyl fluoride (PMSF) were purchased from Sigma-Aldrich (St. Louis, MO, USA). Sodium/potassium tartrate, copper(II) sulfate, and glycerol were purchased from Reagen (Colombo, PR, Brazil). Polyvinylidene fluoride (PVDF) membranes, methanol, dimethyl sulfoxide, and wortmannin were purchased from Merck Millipore (Barueri, SP, Brazil). ECL Prime, sodium dodecyl sulfate (SDS), and Tris were purchased from GE Healthcare (Pittsburgh, PA, USA). LDH Liquiform kit (no. 86-2/30), BUN kit (no. 27–500), and Sensiprot kit (no. 36) were purchased from Labtest (Lagoa Santa, MG, Brazil). Creatinine kit (no. 335) was purchased from Gold Analisa (Belo Horizonte, MG, Brazil). Sodium enzymatic kit (no. 573351) was purchased from In Vitro Diagnóstica (Itabira, MG, Brazil). MK-2206 was purchased from Selleckchem (Houston, TX, USA). Annexin V:FITC apoptosis detection kit I was purchased from BD Biosciences (São Paulo, SP, Brazil). Polyclonal phospho-mTOR (Ser-2481), monoclonal mTOR (clone 7C10), polyclonal phospho-PKB (Ser-473), polyclonal PKB, polyclonal iNOS, polyclonal β-actin and HRP-conjugated anti-rabbit IgG antibodies were purchased from Cell Signaling Technology (Danvers, MA, USA). Polyclonal albumin, monoclonal GSK3β (clone 3D10), polyclonal phosphorylated GSK3β (Ser9) and polyclonal Lrp2/megalin antibodies were purchased from Abcam (Cambridge, MA, USA). Monoclonal F4/80 antibody (clone A3-1) was purchased from AbD Serotec (Raleigh, NC, USA). Monoclonal arginase-1 antibody (clone 19) was purchased from BD Transduction (Mississauga, ON, Canada). Biotinylated anti-rabbit or anti-mouse IgG was purchased from Agilent Technologies (Santa Clara, CA, USA). Dulbecco’s modified Eagle’s medium (DMEM), PBS, 4′,6-diamidino-2-phenylindole (DAPI), fetal bovine serum (FBS), 3,3′-diaminobenzidine (DAB), and UltraPure *N*,*N*′-methylenebisacrylamide were purchased from Thermo Fisher Scientific (Waltham, MA, USA). Vectashield antifade mounting medium was purchased from Vector Laboratories (Burlingame, CA, USA). LLC-PK1 cells were obtained from the American Type Culture Collection (Rockville, MD, USA). All other reagents were of the highest purity available.

### Animals and experimental protocol

Male BALB/C mice (8–11 weeks old), weighing 20–25 g, were obtained from the Institute of Science and Technology in Biomodels (ICTB) of the Oswaldo Cruz Foundation (FIOCRUZ), Rio de Janeiro, Brazil. The animals were accommodated in an air-conditioned environment (22–24°C) in a regular 12-h light/dark cycle with water and standard chow ad libitum. All procedures involving the handling of animals were conducted in accordance with the National Institutes of Health (NIH) Guide for the Care and Use of Laboratory Animals and were approved by the Institutional Ethics Committee of the Federal University of Rio de Janeiro (protocol number 043/18). During the study, the presence or absence of adverse clinical signs associated with the BALB/C strain such as male aggression, corneal opacity, conjunctivitis, blepharitis, or periorbital abscesses was checked. In addition, other possible abnormalities such as skin lesions, occurrence of tumors, hydration status, body condition, and abnormalities in the teeth, genitals, and abdomen were analyzed. Furthermore, general behavior aspects such as degree of mobility inside the cage, interaction with cage mates, eating, drinking, absence of feces or diarrhea, and the ability of the animals to build a nest were also monitored.

The animal model of TII induced by albumin overload was developed as described previously [22,30,31]. Briefly, mice were randomly divided into 4 experimental groups: (1) control group (CONT); (2) BSA-treated group (BSA) subjected to intraperitoneal (i.p.) injection of BSA 10 g/kg/day for 7 consecutive days; (3) simultaneous BSA and lithium-treated group (BSA+LIT), subjected to simultaneous i.p. injection of BSA and lithium administration 300 mg/kg/day via gavage; (4) lithium-treated group (LIT), subjected to lithium administration alone. The CONT and LIT groups received i.p. injections with saline used as vehicle for BSA. From day 5 to day 7 of treatment, the animals were housed in metabolic cages to allow biochemical analysis of urine and other clinical parameters. In order to minimize suffering, the animals were euthanized at the end of day 7 with a combination of the following anesthetics: ketamine (240 mg/kg body weight) and xylazine (15 mg/kg body weight). The kidneys and blood were then collected for different analyses. When indicated, the kidneys were prepared for: (1) histologic and immunohistochemistry studies; (2) protein phosphorylation or protein expression in the homogenate fraction of the renal cortex; or (3) *in vivo* PT albumin reabsorption.

### Analysis of renal function

Analysis of renal function was performed as described previously [22,30–32]. Briefly, the 24-h urine was collected to determine urinary flow (μL/min). Then, the urine samples were clarified by centrifugation at 600 × *g* for 10 min to remove urine sediments, and the supernatant was used to determine the protein, creatinine, and sodium levels. Plasma samples were obtained after centrifugation of the blood samples collected by cardiac puncture (600 × *g* for 2 min). Plasma samples were used to determine creatinine, sodium, and blood urea nitrogen (BUN) levels. All these parameters were determined using commercial kits available from Gold Analisa and Labtest (Brazil). The creatinine levels were determined by the alkaline picrate method. 25 μL of non-diluted plasma or diluted urine (50×) was added to 250 μL of a reaction medium containing picric acid. The levels of urinary protein were determined by the pyragallol red method. BUN levels were measured using the urease method. Sodium levels were determined by precipitation with uranyl-magnesium acetate. The results were used to calculate the creatinine clearance (CCr), urinary protein/urinary creatinine ratio (UPCr), and fractional excretion of sodium (FENa^+^).

### *In vivo* albumin reabsorption assay

The *in vivo* albumin reabsorption assay was carried out as described previously [33]. Briefly, the mice were anesthetized and infused intravenously with a single dose of 5 μg/g BSA-FITC used as a tracer. Other mice were infused with the vehicle (used as a blank). After 15 min of infusion, the mice were perfused with heparinized saline to extract serum contaminants and the non-accumulated BSA-FITC, and the kidneys were removed. Then, the renal cortex was isolated and homogenized in ice-cold Ringer solution (20 mM HEPES-Tris [pH 7.4], 140 mM NaCl, 2.7 mM KCl, 1.8 mM CaCl_2_, 1 mM MgCl_2_, 5 mM D(+)-glucose) containing 1 mM PMSF and protease inhibitor cocktail 1×. The homogenate was clarified twice by centrifugation at 15,000 × *g* at 4°C for 10 min. The supernatant obtained was used to determine the fluorescence intensity accumulated in the kidneys. Fluorescence intensity (excitation = 480 nm, emission = 520 nm) was quantified with SpectraMax M2 (Molecular Devices, Sunnyvale, CA). The BSA-FITC-specific uptake was calculated as the difference between the fluorescence units obtained in the homogenate of BSA-FITC-injected mice and those obtained in the homogenate of blank mice. These BSA-associated fluorescence values were normalized to the total protein concentration in the sample using the Folin phenol method as described previously [34]. Data were expressed as arbitrary units.

### Histologic and immunohistochemistry studies

The histologic and immunohistochemistry studies were performed as described previously [22,30–33]. Briefly, the euthanized mice were perfused with saline and 4% paraformaldehyde using a peristaltic pump at a flow rate of 10 mL/min. The kidneys were removed and fixed in Gendre solution for 24 h, in 10% buffered formalin for 48 h, and then embedded in paraffin. The kidneys were sectioned in different sizes. First, 5-μm-thick sections were stained with periodic acid-Schiff reagent for analysis of the area of cortical interstitial space. These sections were also used to determine F4/80-positive cells and megalin expression through immunohistochemistry assays. Then 8-μm-thick sections were stained with Picrosirius red for analysis of cortical collagen deposition. In the immunohistochemistry studies, the detection of F4/80-positive cells and megalin was performed using specific antibodies according to the manufacturer’s instructions. Biotinylated anti-rabbit or anti-mouse IgG was followed by streptavidin-peroxidase, both for 1 h at room temperature. Positive reactions (brown staining) were revealed with DAB, and the sections were counterstained with Harry’s hematoxylin.

All images from the renal cortex were obtained using a Nikon 80i eclipse microscope (Nikon, Japan). All quantification analysis was performed using Image-Pro Plus image analysis software (Media Cybernetics, Inc., USA) in at least 30 randomly captured photomicrographs. To specifically assess the cortical tubular interstitial area, photomicrographs containing large blood vessels and glomeruli were excluded. Analysis of the area of interstitial space was performed by directly measuring the area among the tubules. Collagen deposition analysis was performed by measuring the density of red fibers. Data were expressed as a percentage of the interstitial area with positive staining. The density of F4/80-positive cells and megalin levels was measured by the area with positive staining. Data were expressed as a percentage of the total tissue area.

### Cell culture

Cell culture procedures were carried out as described previously [18,19,21–23,33]. Briefly, LLC-PK1 cells were maintained in low-glucose DMEM supplemented with 10% FBS and 1% penicillin/streptomycin (37°C and 5% CO_2_) until 95% confluence was reached (typically 2 days after seeding). Then, the cells were washed twice with PBS and incubated with FBS-depleted medium in the presence or absence of albumin and/or lithium under different experimental conditions described in the Results section. When indicated, the cells were pre-incubated with specific inhibitors. Serum starvation was done to keep several signaling proteins triggered by growth factors under a basal state of activation. After treatment, the cells were used in different experimental assays such as albumin endocytosis, protein phosphorylation through immunoblotting, and apoptotic cell rate through flow cytometry [18,19,21–23,33]. Cell viability during different experiments was determined by LDH activity in the cell supernatant via the NADH oxidation method using a commercial kit.

### In vitro albumin endocytosis

*In vitro* albumin endocytosis was measured with a BSA-FITC uptake assay as described previously [21,23,33]. Briefly, the treated cells were washed 3 times with pre-warmed Ringer solution (12.4 mM HEPES-Tris [pH 7.4], 140 mM NaCl, 2.7 mM KCl, 1.8 mM CaCl_2_, 1 mM MgCl_2_, 5 mM glucose), and then incubated with Ringer solution containing 30 μg/mL BSA-FITC at 37°C for 30 min. After the reaction, the unbound BSA-FITC was removed by washing 11 times with ice-cold Ringer solution. Parallel experiments were performed on cells held at 4°C to abolish endocytosis and verify possible non-specific bound BSA-FITC. BSA uptake was assessed by cell-associated fluorescence using albumin-FITC or the fluorescence microscopy method.

In the cell-associated fluorescence method, the cells were lysed using detergent solution (0.1% Triton X-100 in 20 mM MOPS) and the cell-associated fluorescence was measured using a microplate spectrofluorimeter (SpectraMax M2, Molecular Devices, Sunnyvale, CA, USA). BSA-specific uptake was calculated as the difference between the BSA-FITC uptake in the absence and in the presence of 20 mg/mL unlabeled BSA. The blank in the experiment was defined as the fluorescence measured in the absence of BSA-FITC; its value was less than 10% of the total fluorescence. The BSA-specific uptake values were further normalized by the total protein concentration in the samples using the Folin phenol method [34].

For fluorescence microscopy, the cells were fixed with 4% paraformaldehyde at 25°C for 15 min and permeabilized with 0.1% Triton X-100 for 1–2 min. To stain the cell nucleus, the cells were incubated with DAPI. The cells were then mounted in Vectashield medium. To quantify albumin endocytosis, images of fluorescent-labeled markers were acquired with a Leica TCS SP8 confocal microscope using a ×63 oil immersion objective lens, and the average intensity per cell was calculated using Image-Pro Plus software v7.0.1.658 (Media Cybernetics, Rockville, MD, USA) from multiple images after subtracting the background.

### Preparation of the renal cortex homogenate fraction

The renal cortex homogenate fraction was obtained as described previously [30–33]. Briefly, the euthanized mice were perfused with heparinized saline using a peristaltic pump with a flow rate of 10 mL/min, and the kidneys were then removed. The renal cortex was isolated using a microtome and homogenized in a cold solution containing 250 mM sucrose, 10 mM HEPES-Tris (pH 7.6), 2 mM EDTA, and 1 mM PMSF. The homogenate was clarified twice by centrifugation at 15,000 × *g* at 4°C for 10 min. The supernatant obtained was stored at −80°C until use.

### Immunoblotting

Immunoblotting was performed as previously described [18,19,21–23,33]. Briefly, cells were washed 5 times with ice-cold PBS and incubated in ice-cold lysis buffer (20 mM HEPES [pH 7.4], 2 mM EGTA, 1% Triton X-100, 50 mM sodium fluoride, 5 mM sodium orthovanadate, 5 mM sodium pyrophosphate, 10 mM sodium β-glycerophosphate, 1 mM PMSF and 2× protease inhibitor cocktail) for 40 min. The cells were then clarified by centrifugation (4°C for 13 min at 15,000 × *g*). The supernatant was collected and the total protein concentration was quantified by the Folin phenol method [34]. The renal cortex homogenate was obtained and the total protein concentration was determined as described above. Proteins obtained from both the cell lysate and renal cortex homogenate fraction (range, 30–60 μg) were resolved on 9% SDS-PAGE and transferred to PVDF membranes (Millipore Corporation, Billerica, MA, USA), according to the manufacturer's instructions. When indicated, the presence of iNOS, arginase-1, phospho-PKB (Ser473), PKB, phospho-mTOR (Ser2481), mTOR, urinary albumin and β-actin was determined using specific antibodies according to the manufacturer’s instruction. After labeling with primary and HRP-conjugated secondary antibodies, the detection of specific bands was performed using ECL Prime as the substrate for HRP. The images were acquired by chemiluminescence using ImageQuant LAS4000 (GE Healthcare Life Sciences). All images were processed by adjusting the brightness and contrast using NIH ImageJ software (version 1.6.0). This image processing method was applied to every pixel in the original image without changing the information illustrated. The optical density (OD) of the bands was also quantified using ImageJ. In each experiment, the OD related to phospho bands (PKB and mTOR) were normalized to the OD of the total protein bands obtained after stripping and re-probing the same membrane with the corresponding antibodies. The same procedure was performed for the assessment of iNOS and arginase-1 expression. The OD of their respective bands was normalized by the OD of bands related to β-actin.

### Analysis of cell apoptosis

Cell apoptosis was determined by the annexin V-FITC labeling as described previously [18]. Briefly, monolayer cells were released by brief incubation with a trypsin-EDTA solution. Then, 10^5^ cells were resuspended in 1× binding buffer (BD PharMingen) and incubated with annexin V-FITC for 15 min at room temperature in the dark, followed by staining with propidium iodide. Cells were analyzed within 1 h in a FACSCalibur flow cytometer, and Cellquest software (Becton Dickinson) was used to analyze the data. Data are presented as the frequency of early apoptotic cells (annexin-V-FITC-positive cells).

### Statistical analysis

The results are expressed as means ± standard error (SE). GraphPad Prism 7 (version 7, GraphPad Software, San Diego, CA, www.graphpad.com) was used for the statistical analysis. Differences between groups were compared by one-way analysis of variance, followed by the Newman-Keuls post test. Significance was determined as *P* < 0.05.

## Results

### Renal parameters

In the present work, a model of TII induced by albumin overload was used [22,30,31]. The experimental animals were divided into 4 groups as described in the earlier. Table 1 shows the values for body weight, water and food intake, urinary flow, serum creatinine (SCr), urinary creatinine, CCr, BUN, and FENa^+^. We did not observe any changes in body weight, food intake, SCr, CCr, and BUN levels in any experimental group. However, both the BSA+LIT and LIT groups presented higher water intake and urinary flow and decreased urinary creatinine levels compared with the CONT group. The BSA, BSA+LIT, and LIT groups showed an increased level of FENa^+^ compared with the CONT group, indicating modifications in tubular reabsorption.

**Table 1:** Renal function parameters.

	CONT	BSA	BSA+LIT	LIT
Body weight (g)	22.80 ± 0.49	21.20 ± 1.20	21.33 ± 0.88	21.00 ± 0.58
Water intake (mL)	4.80 ± 0.49	4.00 ± 0.32	10.67 ± 3.18*	9.00 ± 1.00*
Food intake (g)	3.63 ± 0.44	2.88 ± 0.23	3.71 ± 0.38	3.93 ± 0.19
Urinary flow (μL/min)	0.70 ± 0.09	0.49 ± 0.06	3.30 ± 1.39*	2.67 ± 0.45*
Serum creatinine (mg/dL)	0.102 ± 0.009	0.102 ± 0.019	0.076 ± 0.001	0.119 ± 0.013
Urinary creatinine (mg/dL)	75.70 ± 15.0	68.85 ± 5.74	34.04 ± 9.80*	17.13 ± 3.11*
Creatinine clearance (mL/min)	0.47 ± 0.06	0.45 ± 0.07	0.55 ± 0.07	0.43 ± 0.08
BUN (mg/dL)	66.83 ± 6.45	65.08 ± 4.66	58.91 ± 7.90	64.16 ± 6.76
FENa^+^	0.138 ± 0.0295	0.233 ± 0.0229*	0.352 ± 0.0498*	0.547 ± 0.103*

The results are expressed as means ± SE. Statistically significant in relation to CONT (*P < 0.05).

### Lithium treatment reduces proteinuria and is correlated to a reduction in albumin reabsorption in PT cells

**Fig 1:**
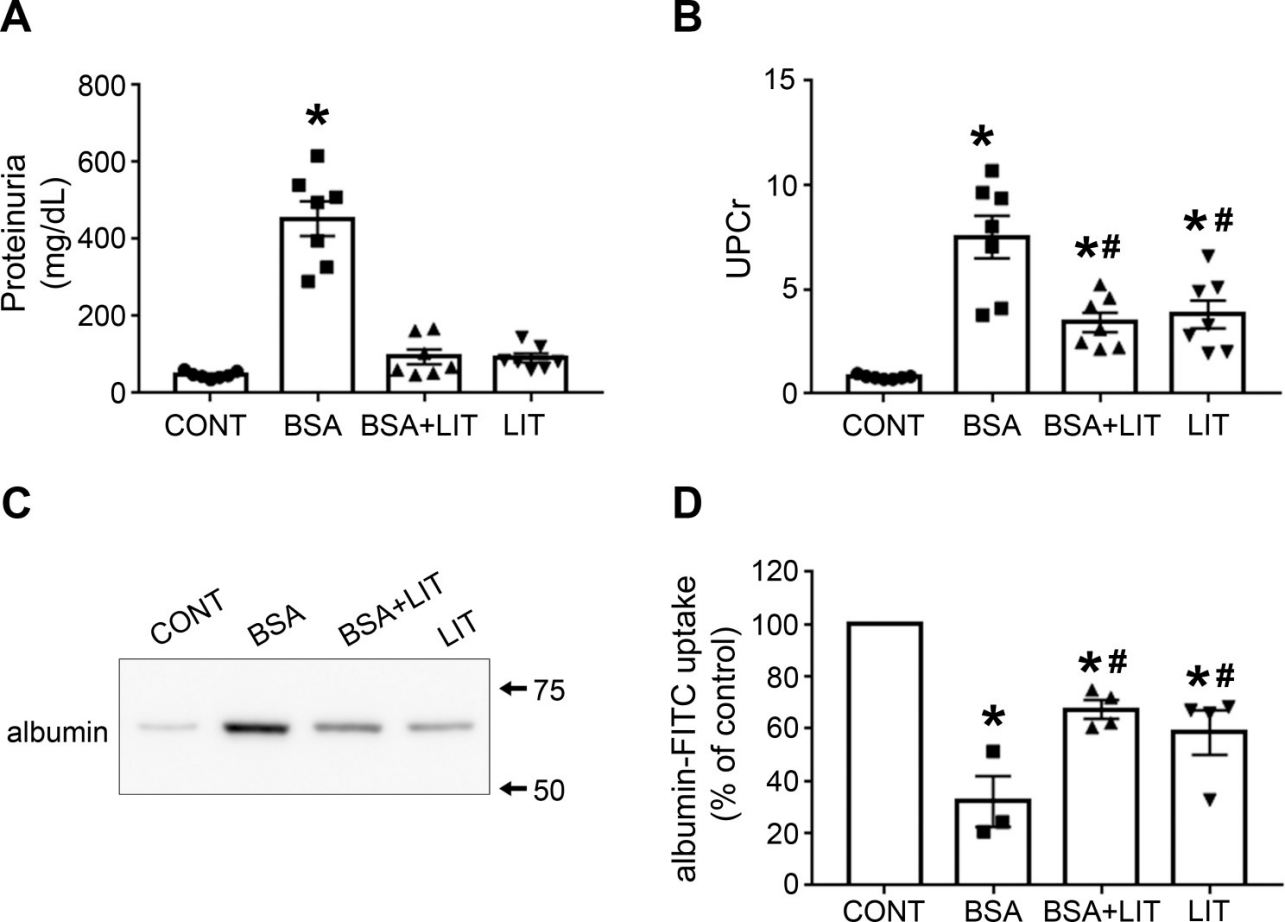
Lithium treatment reduces proteinuria induced by albumin overload due to the increase in albumin reabsorption. Male BALB/C mice were separated into different experimental groups as described in the Materials and methods section. CONT, control group; BSA, group that received intraperitoneal injections 10 g/kg/day BSA for 7 days; BSA+LIT, group that simultaneously received BSA injection and 300 mg/kg/day lithium carbonate via gavage; LIT, lithium-treated group. **A**) Proteinuria (n = 7). **B)** Ratio of urinary protein and urinary creatinine (UPCr; n = 7). **C)** Urinary albumin content was assessed by immunoblotting. Urinary volume was adjusted for 10 μg of urinary creatinine. **D)** *In vivo* albumin reabsorption (n = 4). Mice received a single intravenous dose of 5 μg/g BSA-FITC used as a tracer. After 15 min, the animals were perfused with saline and the BSA-specific fluorescence intensity in the renal cortex was determined. The results are expressed as means ± SE. *P < 0.05 versus CONT group; #*P* < 0.05 versus BSA group.

Because proteinuria is a marker of renal injury, we decided to investigate it in different experimental groups. The level of proteinuria was increased in the BSA group compared with the CONT group (Fig 1A). On the other hand, this effect was completely reversed in the BSA+LIT group. Furthermore, proteinuria did not change in the LIT group. To rule out the possible influence of urinary flow in this parameter, the ratio of urinary protein and urinary creatinine (UPCr) was measured (Fig 1B). A significant increase in UPCr was observed in the BSA group, which was partially reversed in the BSA+LIT group. Interestingly, the LIT group also showed an increased UPCr compared with the CONT group. However, this increase was lower in magnitude than the increase observed in the BSA group. Similarly, the same profile was observed when urinary albumin was assessed by immunoblotting (Fig 1C).

To test if these changes in proteinuria promoted by lithium treatment correlate with changes in tubular protein reabsorption, *in vivo* PT albumin reabsorption was assessed using albumin-FITC as a tracer (Fig 1D). A decrease in albumin reabsorption was observed in the BSA group, which partially reversed by simultaneous treatment with lithium (BSA+LIT group). The LIT group also showed a decrease in albumin reabsorption compared with the CONT group but of lesser magnitude than observed in the BSA group. These results agree with the results for the UPCr, indicating a strict correlation between albumin reabsorption and proteinuria in PT cells.

**Fig 2:**
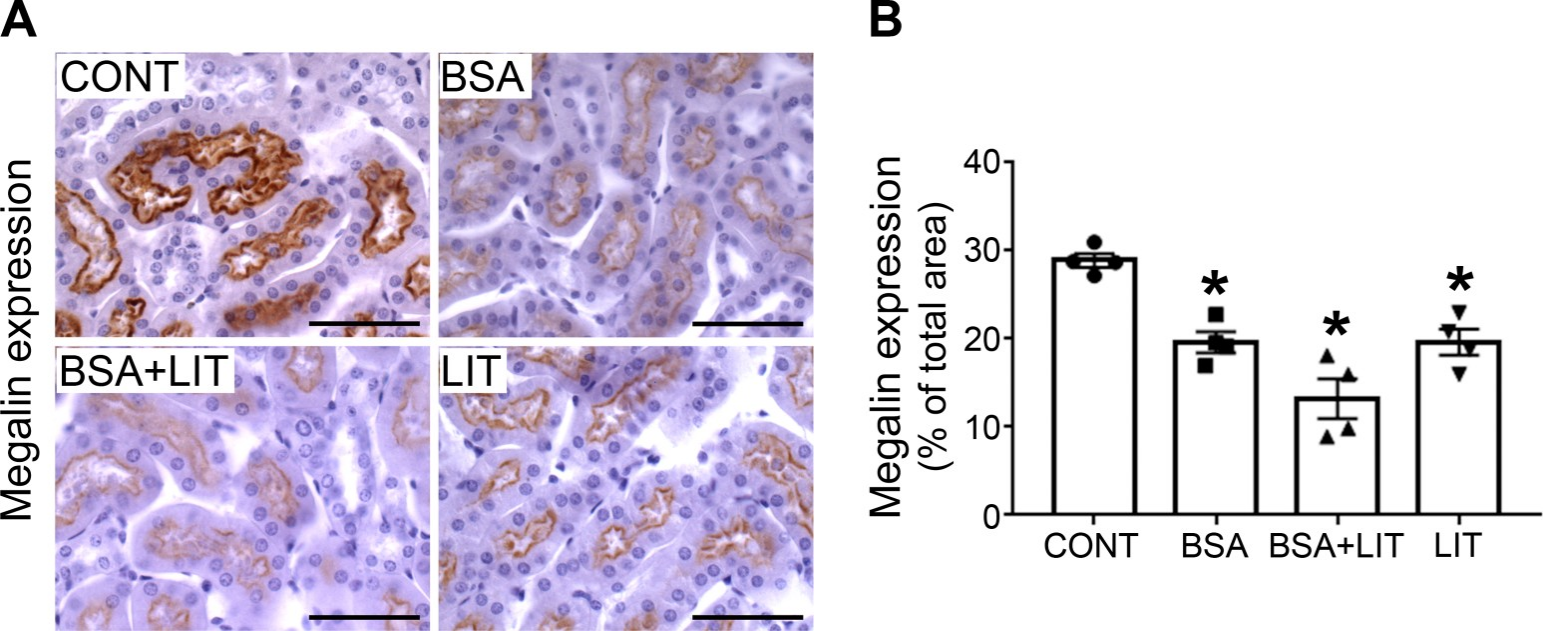
Lithium treatment did not change the inhibition of megalin expression during albumin overload. Mice were treated as described in Fig 1 (n = 4 per group). **A**) Representative megalin staining in the cortical area (bars represent 100 μm). **B**) Quantitative analyses. The results are expressed as means ± SE. *P < 0.05 versus CONT group.

Most of the albumin endocytosis in PT cells occurs through megalin-mediated endocytosis [12,13,17]. In Fig 2, we determined the expression of megalin in the renal cortex. All treated groups (BSA, BSA+LIT, and LIT) presented lower levels of megalin expression than the CONT group (Fig 2A and 2B). These results indicate that the changes in albumin reabsorption observed in lithium-treated animals are not due to changes in megalin expression. But how lithium modulates albumin reabsorption in the TII animal model is still an open matter. One possibility could be an increase in the endocytosis rate rather than endocytosis receptor expression.

### Lithium modulates albumin endocytosis: Involvement of the PI3K/PKB pathway

**Fig 3:**
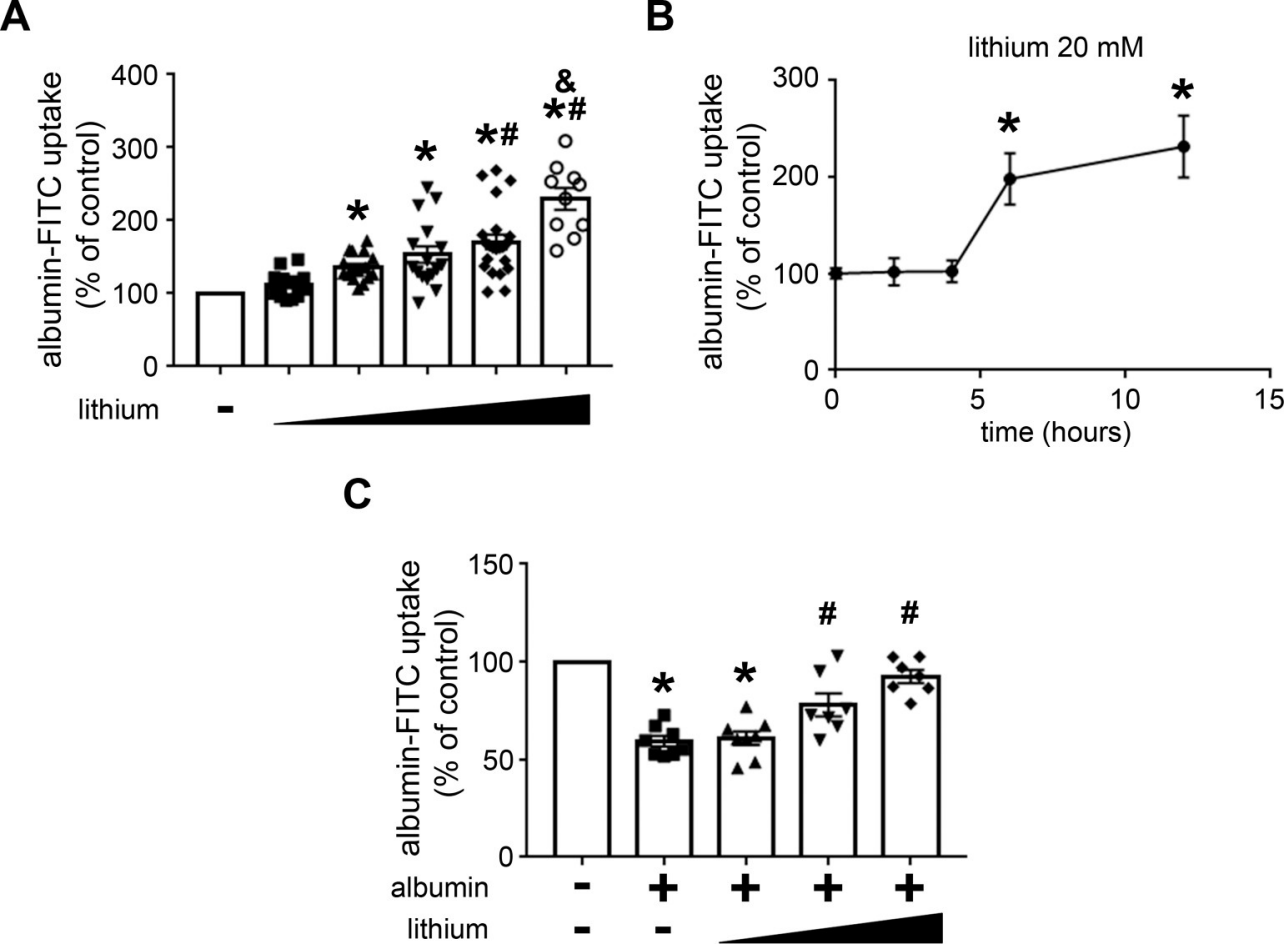
Lithium blockade of the inhibitory effect of high albumin concentration on albumin endocytosis in PT cells measured by cell-associated fluorescence. LLC-PK1 cells were grown in 24-well plates until 95% confluence was reached. Then, the cells were incubated with albumin and/or lithium under different experimental conditions. Albumin endocytosis measured by cell-associated fluorescence using albumin-FITC. **A)** Dose-response effect of lithium on albumin endocytosis (n = 9). The lithium concentrations used were 0.1, 1, 5,10, and 20 mM. **B)** Time-course effect of lithium on albumin endocytosis (n = 4). Times of incubation were 0, 2, 4, 6, and 12 h. **C)** Lithium treatment reversed the inhibitory effect of high albumin concentration on albumin endocytosis (n = 7). The cells were pre-incubated with 20 mg/mL albumin in the absence or presence of increasing lithium concentrations (5, 10, and 20 mM). The results are expressed as means ± SE. A) *P < 0.05 versus control (in the absence of lithium), #P < 0.05 versus 1 mM lithium; &P < 0.05 versus 10 mM lithium. B) *P < 0.05 versus control (in the absence of lithium). C) *P < 0.05 versus control (in the absence of albumin), #P < 0.05 versus albumin.

**Fig 4:**
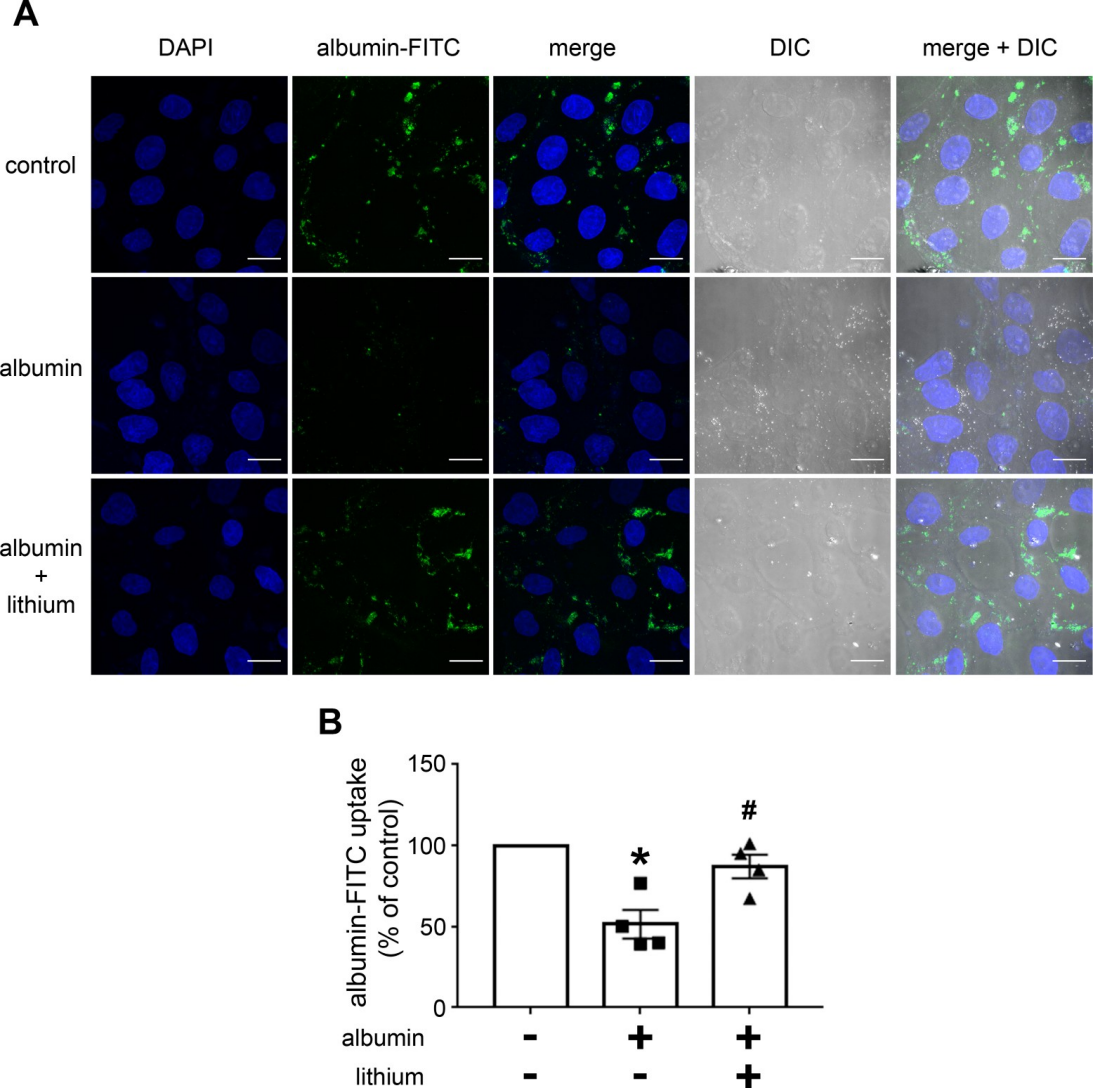
Lithium blockade of the inhibitory effect of high albumin concentration on albumin endocytosis in PT cells measured by confocal fluorescence microscopy. The cells were pre-incubated with 20 mg/mL albumin and/or 20 mM lithium. **A)** Representative image. Green (BSA-FITC) indicates endocytic albumin, and blue (DAPI) indicates the cell nucleus. Scale bar represents 20 μm. DIC, differential interference contrast. **B)** Quantitative analysis (n = 4). The results are expressed as means ± SE. The control was taken as 100%. *P < 0.05 versus control (in the absence of lithium), #P < 0.05 versus albumin.

In this context, it has been proposed that the PI3K/PKB pathway has a central role in the modulation of albumin endocytosis and, consequently, in the development of this renal injury [18,19,21–23]. We decided to study the modulatory effect of lithium on PT albumin endocytosis and the possible involvement of the PI3K/PKB pathway. To address this question, LLC-PK1 cells, a model of PT cells, were used. Initially, the cells were incubated with increasing concentrations of lithium (from 0.1 to 20 mM), and albumin endocytosis was measured after 12 h. Albumin endocytosis was increased with lithium; the maximal effect was obtained at 20 mM (Fig 3A). The time-course experiments showed that this effect was only observed after 6 h of incubation and maintained for up to 12 h (Fig 3B). Figs 3C and 4 show the modulatory effect of albumin on PT albumin endocytosis by lithium. Incubation of the cells with 20 mg/mL albumin for 12 h inhibited albumin endocytosis. Interestingly, this effect was completely reversed when cells were incubated simultaneously with lithium. This result was similar to that obtained in the TII animal model (Fig 1D).

**Fig 5:**
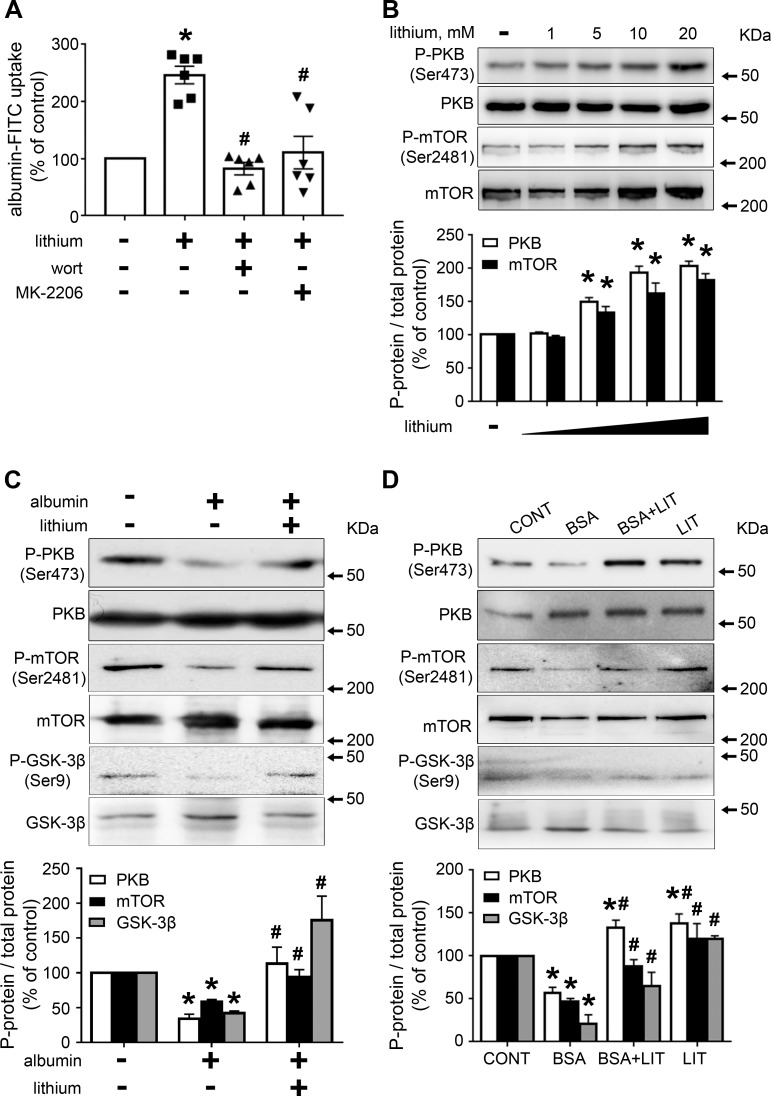
Lithium treatment increases albumin endocytosis due to activation of the mTORC2/PKB/GSK3β pathway. LLC-PK1 cells were grown in 24-well plates until 95% confluence was reached. Then, the cells were incubated with different compounds as indicated. **A)** The stimulatory effect of lithium on albumin endocytosis depends on the PI3K/PKB pathway (n = 6). The cells were pre-incubated with 10−7 M wortmannin (Wort, PI3K inhibitor) or 10−6 M MK-2206 (PKB inhibitor) for 30 min before the addition of 20 mM lithium. **B)** Dose-response of lithium on PKB (Ser473) and mTORC2 (Ser2481) phosphorylation (n = 3). **C)** Lithium treatment modulates the inhibitory effect of higher albumin concentration on PKB (Ser473), GSK3β (Ser9) or mTORC2 (Ser2481) phosphorylation in LLC-PK1 cells (n = 4). **D)** Lithium treatment modulates the inhibitory effect of higher albumin concentration on PKB (Ser473), GSK3β (Ser9) or mTORC2 (Ser2481) phosphorylation in the TII animal model (n = 6). Representative immunoblotting images are shown. Densitometric quantification was obtained as a ratio of phosphorylated and total protein. Relative expression is represented as a percentage of control. The results are expressed as means ± SE. A) *P < 0.05 versus control (in the absence of lithium), #P < 0.05 versus 20 mM lithium. B) *P < 0.05 versus control (in the absence of lithium). C,D) *P < 0.05 versus control (in the absence of albumin and lithium) or CONT group, #P < 0.05 versus albumin or the BSA group.

To investigate the possible role of the PI3K/PKB pathway on the stimulatory effect of lithium on albumin endocytosis, LLC-PK1 cells were pre-incubated with 10^−7^ M wortmannin, PI3K inhibitor, and 10^−6^ M MK-2206, a specific PKB inhibitor. Both inhibitors completely blocked the stimulatory effect of lithium on albumin endocytosis, indicating the role of the PI3K/PKB pathway (Fig 5A). In agreement with this result, it was shown that lithium increased PKB activity measured by phosphorylation of the Ser473 residue (Fig 5B). Because the phosphorylation of Ser473 is mediated by mTORC2, we decided to investigate its possible modulation by lithium treatment [24]. Interestingly, lithium increased mTORC2 activity, measured by phosphorylation of Ser2481, similar to that observed with the phosphorylation of Ser473 at PKB (Fig 5B) [19].

Previously, it was shown that a reduction in PKB activity in the renal cortex is associated with a decrease in albumin endocytosis induced by higher albumin concentration [22]. Fig 5C shows that 20 mM lithium completely reversed the inhibitory effect of 20 mg/mL albumin on the phosphorylation of PKB at Ser473, mTORC2 at Ser2481 and GSK3β at Ser9 in LLC-PK1 cells. Moreover, the phosphorylation of all residues was assessed in the renal cortex of TII induced by albumin overload (Fig 5D). A significant decrease in the phosphorylation of PKB, mTORC2 and GSK3β was observed in the BSA group compared with the CONT group. On the other hand, the simultaneous treatment of the animals with lithium (BSA+LIT group) reversed the inhibitory effect on the phosphorylation of all these kinases observed in the BSA group.

**Fig 6:**
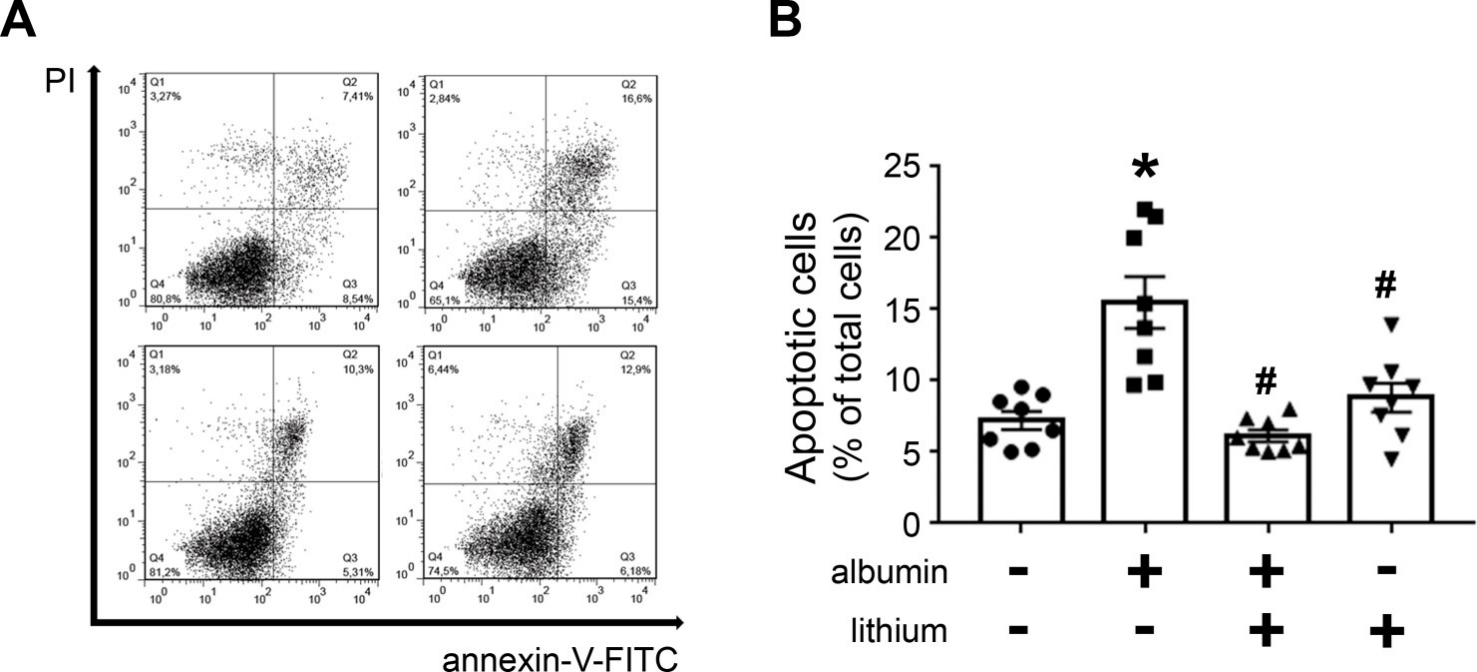
Lithium treatment protects PT cells against apoptosis induced by high albumin concentrations. LLC-PK1 cells were grown in 24-well plates until reach 95% confluence was reached. Then, the cells were incubated with 20 mg/mL albumin and/or 20 mM lithium. **A**) Representative dot plot of the experiments (n = 8). **B**) Quantitative analysis showing the frequency of annexin-V-positive cells. *P < 0.05 versus control (in the absence of albumin), #*P* < 0.05 versus 20 mM albumin.

Usually, modulation of PKB activity is associated with cell death by apoptosis [18,24]. Thus, the possible effect of lithium in apoptosis induced by high albumin concentration in LLC-PK1 cells was tested. Cell apoptosis was determined using annexin V-FITC and flow cytometry. The incubation of LLC-PK1 cells with 20.0 mg/mL albumin overnight increased apoptotic cells, and this effect was completely reversed when the cells were co-incubated with 20 mM lithium (Fig 6A and 6B). Lithium treatment alone did not change the basal rate of cell apoptosis.

So far, our results indicate that the modulatory effect of lithium on the inhibition of PT albumin endocytosis induced by albumin overload involves the activation of PKB, which is mediated by mTORC2 activation. Some evidence shows that changes in PT albumin reabsorption could be a trigger for TII [10,11,22,30,31,35]. Thus, TII was assessed in the next experiments.

### Tubule-interstitial injury induced by proximal tubule albumin overload is attenuated by lithium treatment

**Fig 7:**
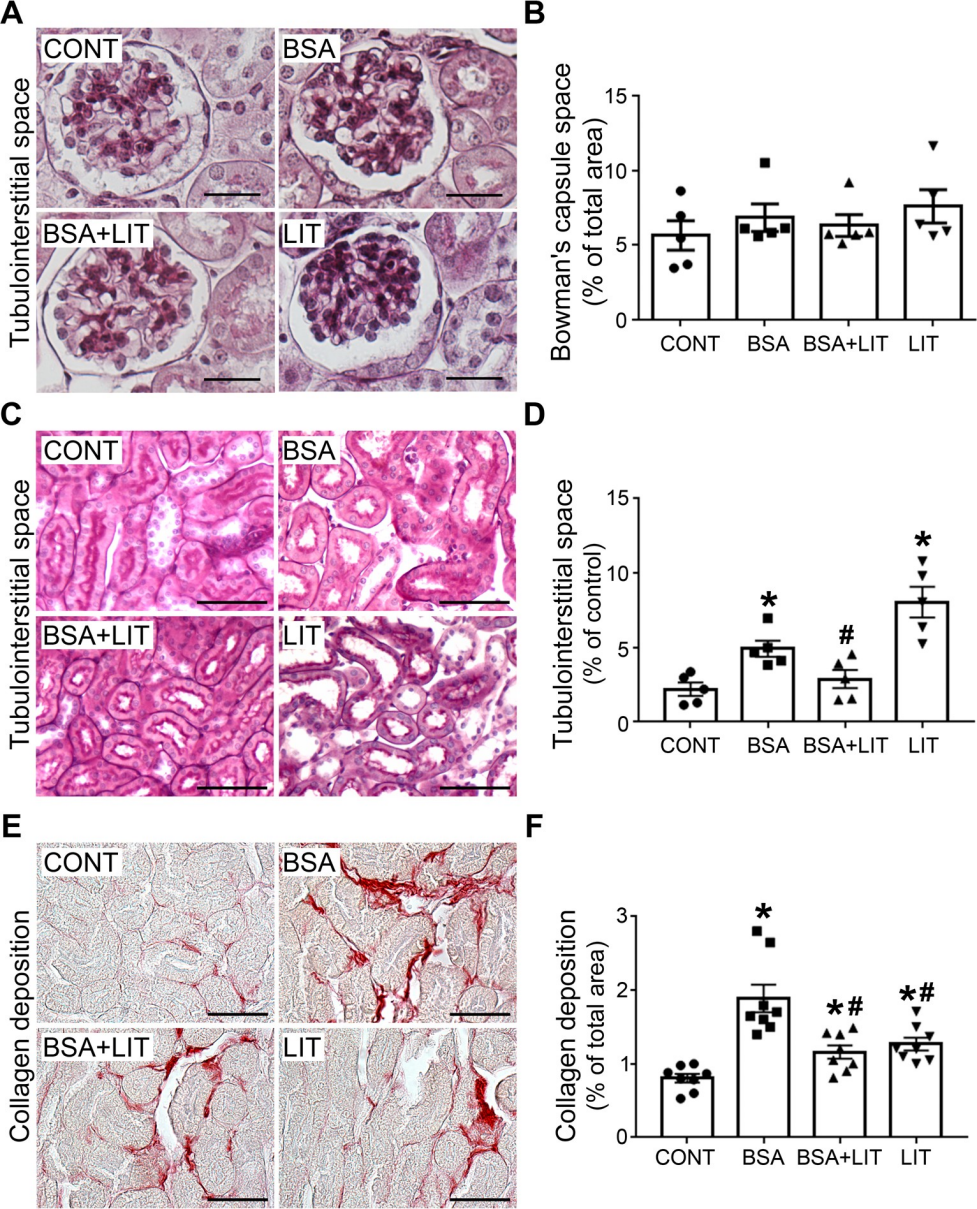
Lithium treatment ameliorates tubule-interstitial injury induced by albumin overload. Mice were treated as described in Fig 1. **A)** Representative images of Bowman´s capsule space. **B)** Quantitative analysis related to A. **C)** Representative images of tubule-interstitial space. **D)** Quantitative analysis related to C (n = 5). **E)** Representative Picrosirius staining (red color) in the cortical area. **F)** Quantitative analysis related to C (n = 8). Scale bars represent 100 μm. The results are expressed as means ± SE. *P < 0.05 versus the CONT group, #P < 0.05 versus the BSA group.

Initially, Bowman´s capsule space was measured (Fig 7A and 7B). This parameter did not change in any experimental conditions, in agreement with the plasma levels of creatinine, BUN and CCr (Table 1). On the other hand, tubule-interstitial space increased in the BSA group and was completely abolished with concomitant lithium treatment (BSA + LIT group; Fig 7C and 7D). Interestingly, lithium treatment (LIT group) alone also increased the area of tubule-interstitial space, similar to the BSA group.

Collagen deposition was increased in the BSA group and was partially reversed by concomitant lithium treatment (BSA+LIT group; Fig 7E and 7F). Lithium treatment (LIT group) alone also increased collagen deposition to a level similar to that observed in the BSA+LIT group. So far, these data suggest that lithium treatment induces a dual effect: (1) tubular damage in healthy mice; and (2) amelioration of TII induced by albumin overload in PT cells. These opposite effects could be associated with differential changes in immune cell infiltration, such as in macrophages.

**Fig 8:**
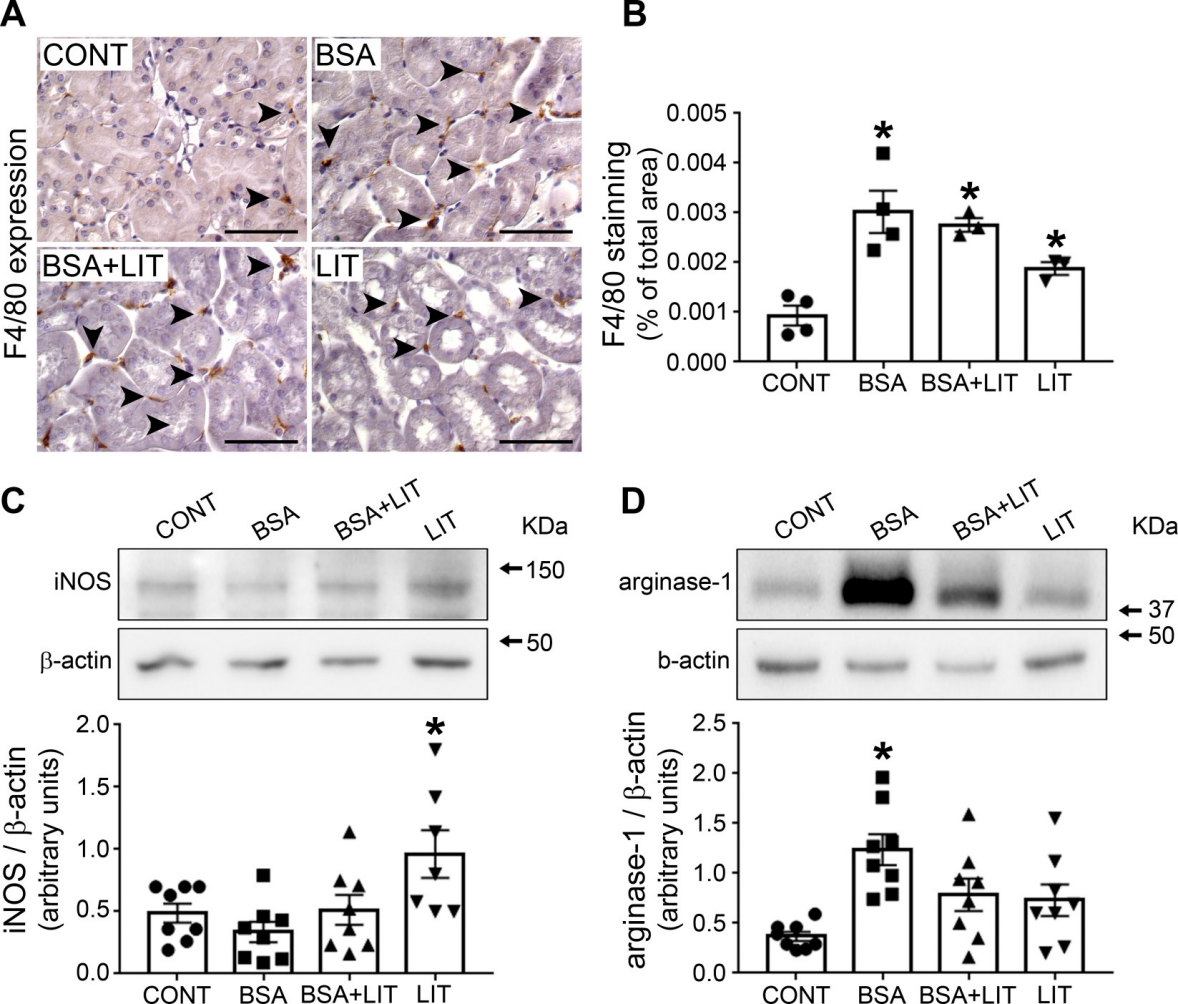
Lithium treatment induces change in M1/M2 phenotype macrophages. Mice were treated as described in Fig 1. **A)** Representative areas of macrophage infiltration by F4/80 staining in the cortical area. Arrows denote positive brown staining. Scale bars represent 100 μm. **B)** Quantitative analysis related to E (n = 4). **C)** iNOS expression (n = 8). **D)** Arginase-1 expression (n = 8). Both iNOS and arginase-1 bands were detected using specific antibodies. The OD related to iNOS or arginase-1 bands was normalized to the OD related to the β-actin band. The results are expressed as means ± SE. *P < 0.05 versus the CONT group.

Fig 8A and 8B shows the extent of macrophage infiltration by measuring the presence of F4/80-positive cells in the renal cortex. Interestingly, an increase in macrophage infiltration was observed in all treated groups (BSA, BSA+LIT, and LIT) compared with the CONT group. This lack of influence of lithium on macrophage infiltration triggered by albumin overload seems to be contrary to the protective effect of lithium on TII induced by albumin overload, suggesting another possible effect of lithium, such as modulation of the macrophage phenotype.

To test this hypothesis, iNOS and arginase-1 expression (markers of M1 or M2 subtype macrophages, respectively) were determined in the renal cortex. We observed that iNOS expression was only increased in the LIT group (Fig 8C). On the other hand, arginase-1 expression was increased in the BSA group, and this effect was reversed by simultaneous lithium treatment (BSA+LIT group; Fig 8D). The level of arginase-1 expression was not changed in the LIT and BSA+LIT groups compared with CONT group despite a tendency to be higher. These results indicate that changes in the balance of M1/M2 phenotype macrophages are associated with the effect of lithium on TII induced by albumin overload.

## Discussion

Despite the widespread use of lithium for the treatment of mood disorders, the renal effects are still controversial [2–8]. Some studies indicate that lithium treatment ameliorates AKI induced by LPS or IR, and this effect is correlated to injuries in cortical nephron segments [6–8]. We studied the effect of lithium treatment on TII induced by albumin overload. It was observed that development of TII was attenuated by lithium treatment. We are proposing that this effect involves modulation of PT albumin endocytosis and activation of the mTORC2/PKB pathway.

One important concern could be correlated to the dose of lithium used. Some studies have used a lower dose of lithium chloride (30–80 mg/kg), by intraperitoneal injection, as an inhibitor of GSK-3β to treat renal diseases in LPS-induced or gentamicin-induced AKI model mouse models [6–8]. In the present study, male BALB/C mice were treated with 300 mg/kg/day lithium carbonate by gavage. This dose was chosen because it mimics the clinical dose used to treat mood disorders [36].

For several decades, albuminuria has been used as a marker of progression of renal disease. More recently, it has been proposed that albumin overload in PT promotes TII, and, consequently, induces progression of renal disease to end-stage renal disease [9–13]. Some authors, including our group, showed a correlation with albumin overload in PT cells, impairment in PT albumin reabsorption, and the development of an inflammatory phenotype [20,22,30,31,35]. In this context, changes in albumin endocytosis in PT cells is a crucial process to determine both albumin reabsorption and the development of TII.

It has been shown that cubilin assembles with megalin and amnionless, forming a receptor involved in PT albumin reabsorption [13,37]. However, some authors have proposed that this complex does not work in the same way for all protein reabsorption in PT cells [38]. It was observed that cubilin knockout mice do not have significant proteinuria even though they present mild albuminuria. On the other hand, specific PT cells in megalin knockout mice have high proteinuria and albuminuria [38]. The aim of the present work was to correlate megalin expression, PT albumin reabsorption and albuminuria. However, the role of cubilin in the albuminuria observed under the different experimental conditions cannot be ruled out.

It was shown that lithium treatment reduced albuminuria and proteinuria in animal models of renal disease, such as lupus and adriamycin-induced kidney injury [14–16]. In agreement, we also observed that lithium treatment ameliorated the inhibitory effect of high albumin concentrations on PT albumin endocytosis in both *in vivo* and *in vitro* models, which did not involve modulation of the albumin receptor megalin. One possibility to explain this effect could be an increase in the rate of albumin endocytosis induced by post-translation modifications due to activation of regulatory signaling pathways.

Several studies have pointed out the correlation between PKB activity and albumin endocytosis [18,19,21–23]. It has been well accepted that the PI3K/PKB pathway is a central pathway to modulate PT albumin endocytosis. Its activation leads to an increase in albumin endocytosis, whereas its inhibition promotes the inhibition of albumin endocytosis [21,23]. On the other hand, PKB activity could also be modulated by changes in albumin concentration, leading to modifications in cell survival [18]. A higher albumin concentration decreases megalin expression, PKB activity, and induces apoptosis promoting cell death. This mechanism was proposed to be involved in the genesis of TII induced by albumin overload [22]. Here, we observed that lithium treatment prevented the inhibition of PKB activity by albumin overload without changes in megalin expression. Indeed, we observed that lithium treatment per se increased PKB phosphorylation at Ser473. These results indicate that the effect of lithium treatment on albumin endocytosis is due to the increase in PKB activity. In agreement with this proposal, it was observed that the effect of lithium on albumin endocytosis in LLC-PK1 cells is abolished by inhibitors of PKB and PI3K.

Modulation of PKB activity by lithium has been observed in different cell types [25–29]. PKB activation induced by lithium is associated with inhibition of apoptosis, and it is proposed to be involved in the neuroprotective effects of lithium. However, the mechanism involved in the activation of PKB is still an open matter. Mora et al. [27] proposed that lithium increases PKB activity due to the inhibition of protein phosphatase-2A induced by ceramide in cerebellar granule cells. In the present work, we observed that lithium increased mTORC2 activity in LLC-PK1 cells, which is responsible for the phosphorylation of PKB at Ser473 and, consequently, its activation. Furthermore, we observed that lithium protects against apoptosis induced by higher albumin concentration in LLC-PK1 cells. Accordingly, it was observed that lithium treatment reduces apoptosis in cortical tubular segments in an AKI model induced by LPS injection [6].

A possible target downstream of mTORC2/PKB pathway activation could be GSK3β [2,5,6]. It has been described that phosphorylation of GSK3β at Ser9 by PKB leads to its inhibition [24]. Here, we observed the modulation of the phosphorylation of GSK3β at Ser9 by lithium following the same behavior as that observed on the phosphorylation of PKB at Ser473 and mTORC2 at Ser2481. These results indicate that there is a possible correlation among mTORC2, PKB and GSK3β activities. Furthermore, Yussef et al. [39] observed that the treatment of MDCK cells for 4 hours with 50 mM lithium decreases megalin expression on the plasma membrane. It is possible to postulate that lithium treatment ameliorates PT albumin endocytosis due to activation of the mTORC2/PKB pathway, which leads to inhibition of GSK3β and, consequently, the increase in PT albumin endocytosis.

TII induced by albumin overload is associated with secretion of the chemokine MCP-1 by renal cells and macrophage infiltration in the renal cortex [22,31,40,41]. We have not observed any effect of lithium treatment on renal cortex macrophage infiltration induced by albumin overload. Usually, macrophages under activation are driven to M1-subtype (iNOS-positive cell, a pro-inflammatory phenotype) or M2-subtype (arginase-1-positive cell, a resolution phenotype) cells [42–44]. In this context, we observed significant detection of arginase-1, suggesting the possible involvement of M2-subtype macrophages rather than the M1 subtype in TII induced by albumin overload. This idea is supported by the observation that under persistent tissue damage, M2-subtype macrophages contribute to the development of fibrotic processes [45]. In agreement, we observed an increase in collagen deposition during albumin overload in PT cells. This result agrees with the previous observation that TGF-β is increased in an animal model of TII induced by albumin overload [22,46].

Lithium treatment per se induced renal injury despite the beneficial effect of attenuating the development of TII induced by albumin overload. Interestingly, our data, together with those previously published by other authors, indicate that lithium treatment for short periods did not change CCr and BUN, markers of glomerular flow rate [6–8]. However, a possible effect of lithium on the permeability of glomerular protein cannot be ruled out. Dai and coworkers [47] showed that a single-dose lithium chloride treatment (intravenous injection of 16 mmol/kg) of BALB/c mice induced transient albuminuria correlated to podocyte foot process effacement. Furthermore, it has been shown that chronic lithium treatment of patients with bipolar disorders can cause glomerular diseases such as glomerulosclerosis [2]. In a recent work, Hurcombe et al. [48] showed that GSK3 is a critical regulator of podocyte biology and, consequently, of glomerular protein permeability. They observed that treatment of Wistar rats with lithium for 6 months induced inhibition of GSK3 with development of proteinuria and glomerulosclerosis. Here, we observed that lithium increases albumin uptake in LLC-PK1 cells, indicating that proteinuria and albuminuria induced by lithium treatment could not be due to changes in protein tubular endocytosis. Thus, it is plausible to postulate that the proteinuria and albuminuria observed in mice treated with lithium alone could be correlated to an increase in glomerular protein permeability.

On the other hand, lithium decreased megalin expression and albumin endocytosis despite increasing PKB activity in an *in vivo* model. These data indicate that lithium, under the conditions used in the present work, has a specific tubular effect. But how does lithium induce tubular effects? We observed that lithium increased albumin endocytosis in LLC-PK1 cells, indicating that its inhibitory effect observed under *in vivo* conditions could be a consequence of changes in cells surrounding PT cells rather than directly in PT cells. In agreement with this view, it was observed that lithium treatment decrease M2-subtype macrophages induced by albumin overload (BSA+LIT group) and increased M1-subtype macrophages when the animals were treated only with lithium (LIT group). Interestingly, in isolated human macrophages, it was observed that lithium induces the activation of a CCL2 and tumor necrosis factor α secreting subtype macrophage [49–51]. These results together point out a possible role of lithium in modulating macrophage polarization, but further experiments are necessary to clarify this issue.

Evidence indicates that lithium treatment could have a beneficial outcome in AKI of various causes [6–8]. Supporting this view, our results show that lithium decreases the development of TII induced by albumin overload and, consequently, the progression of renal disease. These results indicate that lithium could be used in the treatment of AKI, avoiding progression to a chronic kidney disease (CKD). On the other hand, chronic lithium treatment has been reported to cause kidney damage [2,4]. Thus, its use could be restricted to the treatment of CKD rather than treatment of AKI.

### Conclusion

Our results indicate that the effect of lithium treatment on renal function depends on the conditions used: lithium treatment could have beneficial effects when used to treat renal disease or it could cause side effects on renal function when used to treat non-renal diseases. Its beneficial effect could be correlated to a modulatory effect on the machinery involved in PT albumin reabsorption.
